# Knowledge, Attitudes, and Screening for Obstructive Sleep Apnea and Diabetes Mellitus among War Veterans Seeking Treatment of Posttraumatic Stress Disorder

**DOI:** 10.3390/healthcare9121698

**Published:** 2021-12-08

**Authors:** Ante Mayer, Maja Mizdrak, Marija Babić, Tonći Mastelić, Trpimir Glavina, Joško Božić, Tina Tičinović Kurir

**Affiliations:** 1 Health Centre of Split-Dalmatia County, 21000 Split, Croatia; mayerante@gmail.com; 2 Department of Nephrology and Hemodialysis, University Hospital of Split, 21000 Split, Croatia; marija-babic92@hotmail.com; 3 Department of Pathophysiology, University of Split School of Medicine, 21000 Split, Croatia; josko.bozic@mefst.hr (J.B.); tticinov@mefst.hr (T.T.K.); 4 Department of Psychiatry, University Hospital of Split, 21000 Split, Croatia; toncimastelic@hotmail.com (T.M.); tglavina0@gmail.com (T.G.); 5 Department of Endocrinology, Diabetes and Metabolic Disorders, University Hospital of Split, 21000 Split, Croatia

**Keywords:** obstructive sleep apnea, STOP-Bang questionnaire, posttraumatic stress disorder, diabetes mellitus, prediabetes, war veterans, screening, cardiometabolic continuum, prevention, education

## Abstract

Posttraumatic stress disorder (PTSD) is one of the most common psychiatric disorders. However, we should not neglect the somatic aspects of PTSD. Associations with cardiovascular diseases (CVD) are particularly concerning because PTSD was associated with an even 53% higher risk for CVD. This study aimed to analyze the prevalence of several CVD risk factors, especially diabetes mellitus among PTSD patients divided into three groups according to obstructive sleep apnea (OSA) risk stratification (low, intermediate, and high). This cross-sectional study included one hundred male PTSD veterans. The mean age was 53 (40–67) years. The estimated OSA risk was 95% for the whole cohort, and 53% were in the high-risk group. Median HbA1c was 5.6 (4.6–10)%. The hemoglobin A1c (HbA1c) levels showed that 34 patients were in the prediabetes group, and 20 of them fulfilled the criteria for diabetes. However, only 13 of them were aware of their previous diagnosis of diabetes mellitus. In testing knowledge about diabetes, 62% and only 23% of patients knew the correct definition of HbA1c and level of fasting plasma glucose, respectively. Diabetic patients had insufficient knowledge about diabetic complications and treatment. A higher level of PTSD symptoms in veterans was associated with a higher prevalence of OSA. The results strongly support further research and education into early detection of CVD risk factors associated with PTSD.

## 1. Introduction

Posttraumatic stress disorder (PTSD) is a mental disorder characterized by persistent and intrusive re-experiencing of traumatic events, an avoidance of reminders of the trauma, and highly elevated levels of anxiety, leading to significant functional impairment [1]. It develops in some people who have experienced exceptionally threatening or horrifying events. PTSD is one of the most common psychiatric disorders among war veterans, affecting approximately 30% of Vietnam veterans and 11% to 17% Iraque and Afghanistan veterans [2,3]. In Croatia, PTSD is diagnosed in circa 15% of veterans, with reported rates ranging up to 40% according to different studies [4,5]. The diagnosis of PTSD is set and treated by mental health experts. However, we should not neglect the somatic aspects and consequences of PTSD. Almost twenty years ago, McEwen and Stellar used PTSD as an example to describe how psychological trauma and repeated reminders of traumatic events cause a cascade of harmful neuronal, hormonal, and immunologic effects [6]. Associations with cardiovascular diseases (CVD) are particularly concerning [7]. Despite advances in prevention and treatment, CVD remains the leading cause of mortality, morbidity, and costs [8]. PTSD was associated with an even 53% increased risk for incident cardiac events or cardiac-specific mortality [7]. Ninety percent of CVD are preventable by respecting risks factors modification, such as smoking cessation, alcohol abuse prohibition, healthy diet, and physical activity. Besides traditional CVD risks factors, recent studies have highlighted another, highly prevalent, but largely underdiagnosed risk factor: obstructive sleep apnea (OSA) [9].

OSA prevalence is continuously increasing, affecting one-third of the male population and one-fourth of women in the United States [9,10]. Data from the literature have linked OSA to multiple cardiovascular comorbidities: hypertension, diabetes mellitus, coronary artery disease, heart failure, and cardiac arrhythmias [9]. In this cross-sectional study, we examined the relationship between PTSD in the Croatian population of homeland war veterans and the risk of obstructive sleep apnea, diabetes mellitus, and several other cardiovascular risk factors. This study aimed to analyze these comorbidities according to their OSA risk status. We have also examined their knowledge about chronic therapy and diabetes control.

## 2. Materials and Methods

### 2.1. Study Population

This study included a cohort of 100 homeland war veterans who sought PTSD treatment at a Trauma Recovery Program for more than one year in the University Hospital of Split, Split, Croatia, from April 2018 to May 2018. This cross-sectional study included all male patients with PTSD diagnoses. Female veterans did not participate due to their small number. Recruitment methods were clinical referral measurements taken by the physicians, completed self-report questionnaires, and medical data examination. All participants provided voluntary informed consent. The study was conducted according to the guidelines of the Declaration of Helsinki, and approved by the Ethics Committee of the University Hospital of Split.

### 2.2. Measures

Body height and weight were measured using an altimeter and a calibrated scale (Seca, Birmingham, UK). The body mass index (BMI) values were used to classify participants as underweight (BMI < 18.5), normal weight (18.5 ≤ BMI < 25.0), overweight (25.0 ≤ BMI < 30), and obese (BMI ≥ 30.0) kg/m^2^. Anthropometric measurements were measured using a tape measure. Daily repeated blood pressure at 9 o’clock a.m. higher than 135/85 mmHg was considered elevated. Hyperlipidemia was defined as abnormally high levels of low-density lipoprotein (>1.4 mmol/L for very high-risk patients; >1.8 mmol/L for high-risk patients; >2.6 mmol/L for intermediate-risk patients and >3.0 mmol/L for low-risk patients), total cholesterol (>5.0 mmol/L), and/or triglyceride levels (>1.7 mmol/L).

Hemoglobin A1c (HbA1c), as the primary outcome measure for glucose control, was measured for each patient. The definition of prediabetes was HbA1c level more than 5.7% and less than 6.5%, while diabetes was diagnosed with an HbA1c level higher than 6.5% according to ADA standards [11]. A blood glucose test was performed in the morning on the day when veterans completed the questionnaire. They were also asked if they ate or not before the test.

### 2.3. Questionnaires

Each participant fulfilled questionnaires prepared by the authors. The first set of questions referred to general data (sex, age, employment, incomes); the other group of questions was about physical activity and relaxing methods; and the third one asked about duration of PTSD, chronic therapy, course of PTSD, and associated comorbidities.

Obstructive sleep apnea (OSA) risk was assessed using the STOP-Bang questionnaire to identify individuals that were at increased risk of having sleep apnea (Appendix A). The questionnaire had eight questions with yes or no answers [12,13]. According to obtained results, patients were divided into three groups due to estimated OSA risk: low risk of OSA—Yes to 0 to 2 questions, intermediate risk of OSA—Yes to 3 to 4 questions, and high risk of OSA—Yes to 5 to 8 questions.

### 2.4. Statistical Analysis

Statistical analysis was performed using National Council for the Social Studies (NCSS) 2021 software. Statistical significance was set at *p* < 0.05. The normality of data distribution was estimated with the Kolmogorov–Smirnov test. Quantitative data were expressed as median and range, while qualitative data were expressed as whole numbers and percentages. Analysis of the statistical significance of differences in several numerical variables was performed with the Kruskal–Wallis test and of differences between the two groups with the Mann–Whitney U test. Categorical variables were analyzed using the chi-square test. Lastly, multiple logistic regression analyses adjusted for age, gender, and anthropometric measurements were used to determine significant independent predictors of the positive STOP-Bang test.

## 3. Results

### 3.1. Basic Anthropometric and Related Characteristics of the Study Sample

In this pilot study, there were 100 male war veterans with the diagnosis of PTSD. The mean age of participants was 53 years, with a range of 40 to 67 years. According to the STOP-Bang questionnaire results, all participants were divided into three groups. The first one represents the low risk of OSA, the second represents those of intermediate risk, and the third group represents classified veterans with an estimated high risk of OSA. In the first group, there were only five participants (5%), meaning that 95% of veterans had positive STOP-Bang results. The most common symptom was tiredness (85%), but also very often snoring (62%) and apnea (30%). More than half of them (53%) had a high risk of obstructive sleep apnea. Table 1 showed anthropometric variables of all participants which did not differentiate between the three groups (all *p* > 0.05). Only six participants had normal body mass index, none of them were underweight, and 94% of them were overweight or obese.

### 3.2. War Veterans’ Attitudes toward PTSD and Quality of Life

All of them believed that their diagnosis was strongly associated with homeland war. The average duration of PTSD was 11 years (min–max: 1–25). One-third of the participants (31%) did not know which and how they take medications, 44 knew little about chronic therapy, while one fourth of participants knew all their medications. On average, they were taking three medications. Most commonly used was diazepam (52%), then alprazolam (24%), and promazine (14%). Less than six participants took other drugs. Table 2 shows analyzed parameters based on material condition, working status, and quality of sleep, according to OSA risk stratification. Working status did not correlate to OSA risk (*p* = 0.746), either to material condition (*p* = 0.832). All of them attended group therapy, while 73% also attended individual therapy. According to an assessment of physical activity, we had the most commonly trimodal distribution between 2–3 times per week (28%), every day (19%), and non-active (12%). Physical inactivity did not correlate to higher OSA risk in this study (*p* = 0.827).

Among those who have answered positively to the possibility of relaxing, the most common methods were silence, peace, and loneliness (38.88%); physical activity and gardening (22.22%); and spending time with friends who were not warring veterans (18.52%). Other methods, including medications, hiking and nature, alcohol, fire, lying and sleeping, pets, sexual activity, spending time with war companies, football, music, painting, and reading, were rarely mentioned. Twenty-six participants were hospitalized because of PTSD, on average thrice. The number of hospitalizations due to PTSD was not associated with OSA risk (*p* = 0.862). No connection between head trauma and depression (defined with yes or no as previously set diagnosis) was found (*p* = 0.890).

### 3.3. Prevalence of Diabetes Mellitus and Other Cardiovascular Risk Factors

In further analysis, HbA1c was measured for every participant. Furthermore, 13 participants were already diagnosed with diabetes mellitus, while 21 had a positive family history of DM. During our check, we have found 20 participants with diabetes according to HbA1c level and even 34 participants suspected to have prediabetes. When analyzing diabetic status according to OSA risk, most patients with prediabetes and all with DM were in the group of high OSA risk (Table 3).

By analyzing HbA1c levels, our results have shown that there were statistically significant differences among three groups. The highest HbA1c levels were measured in the intermediate OSA risk group. HbA1c levels were higher in group 2 versus group 3 (*p* = 0.039) and borderline significance between groups 1 and 3 (*p* = 0.064). There was no difference among groups 1 and 2 (*p* = 0.970). Table 4 showed other comorbid diseases in an examined cohort. The most prevalent diagnosis was arterial hypertension, present in 35 participants, but predominately in the group with high OSA risk.

In further screening, vice versa analyses were performed. STOP-Bang scores were analyzed according to the most prevalent comorbidities—diabetes mellitus, arterial hypertension, or depression. For the presence of any of these three comorbidities, significant results were obtained meaning higher STOP-Bang score (*p* < 0.001, *p* < 0.001, and *p* < 0.001, respectively). The logistic regression model has shown the predictive role of DM, arterial hypertension, and depression for obstructive sleep apnea (χ2 = 12.897, *p* = 0.005). STOP-Bang score did not correlate with the duration of PTSD or diabetes mellitus, which accounted approximately for 9.4 years (95% CI 4.55–14.22) (*p* = 0.249 and 0.626, respectively).

### 3.4. Knowledge about Diabetes Mellitus

In an analysis of only previously diagnosed participants with diabetes mellitus (N = 13), the average age was 56.5 years (min–max: 40–67 years). In addition, 23 percent of participants suffered from type 1 DM, 46 percent from type 2, and one-third of participants did not know what type of diabetes they had. The average HbA1c for this population was 8% (CI 95% 6.76–9.25), the minimum was 6.3%, while the maximum was 10%. Fasting plasma glucose levels were less than 11.1 mmol/L in three participants (23.1%), less than 7.1 mmol/L in six participants (46.2%), less than 5.6 mmol/L in three participants (23.1%), and less than 3.6 mmol/L in one participant (7.7%).

In testing knowledge about diabetes mellitus, 62% and only 23% of participants knew the right definition of HbA1c and level of fasting plasma glucose, respectively. One-fourth of participants thought that the normal fasting plasma glucose (FPG) level was <11.1 mmol/L. According to our results, 92% of participants named a doctor as a source of information and, for none of them, the source was friends/family or other patients. Fifteen per cent of participants stated television/radio and the Internet as a source of education. Twenty-three per cent of participants had a perception of DM as a mild disease, the same as the worst possible disease. Half of them were aware that DM can have serious complications, but the same percentage of participants did not know about adverse effects. An assessment of self-knowledge about DM, from 1 to 5, was grade 3 for two-thirds of participants, and none of them graded themselves with 5 (the best grade). To improve disease control, most of the participants mentioned the change of lifestyle, but one-fifth of participants stated more frequent doctor visits, better medications, and more education. All participants, on average, had three episodes of hypoglycemia, but more than a third of them did not know what to do on that occasion.

## 4. Discussion

The results of our pilot study have shown that the south Croatian population of homeland war veterans with PTSD diagnosis have high prevalence of several cardiometabolic risk factors and insufficient knowledge about their comorbidities. According to our results, even 95% of them have positive STOP-Bang results, and more than a half have a high risk for obstructive sleep apnea. In our study, 54% of participants, predominately those with higher OSA risk, have prediabetes or diabetes. It is important to mention that the participants were not aware of these diagnoses. Furthermore, 46 and 48 of them were overweight and obese, respectively. There were more than half smokers, one third suffered from arterial hypertension, and one fourth of hyperlipidemia. Other cardiovascular risk factors were present less frequently. To our best knowledge, this is the first study screening comprehensively several cardiometabolic risk factors and analyzing knowledge and attitudes about the disease in the population of European veterans with PTSD.

The overall OSA prevalence ranged from 3% to 49% in different studies. It was higher in males, obese patients, smokers, and the older population, where it is estimated to be 90% in males, and, among war veterans, it accounted for approximately 75.7% [1,14,15,16,17,18,19,20,21]. In a group of the active-duty military with PTSD, 67.3% were diagnosed with OSA using polysomnography at multi-site military medical centers [22]. However, due to methodological differences, it might be improper to compare results from other similar studies. We used a self-report measure to determine the presence of risk for OSA, which very likely identified more individuals than would have a diagnosis of OSA. The STOP-Bang questionnaire is an easy-to-use and accurate screening tool for OSA risk stratification in the general population. It has a correct probability to predict high-risk patients for OSA compared to the Epworth sleepiness scale [12].

A review of more than 4 million electronic medical records indicated that, among veterans, there was a strong association between OSA and stroke, cardiovascular diseases, hypertension, obesity, insulin resistance and diabetes, depression, sleepiness-related accidents, cancer, and increased mortality [23,24]. According to the World Health Organization, cardiovascular diseases were the killer number 1. They account for approximately 17.5 million deaths globally and posttraumatic stress disorder is associated with the accelerated progression of coronary heart disease [25]. OSA impacts cardiovascular health by metabolic perturbations occurring when repetitively attempting to breathe against an occluded airway. It precipitates nightly episodes of hypoxia, sleep disturbance, and sympathetic nervous system surges through the effects of catecholamines on the heart, vasculature, and platelet function, resulting in arterial hypertension and tachycardia, endothelial dysfunction, systemic inflammation, and insulin resistance [9,26]. According to data from the literature, there are also sex differences in the association of PTSD symptoms and subclinical atherosclerosis. In men, increased PTSD symptoms may increase CVD risk by increasing sympathovagal balance and aortic stiffness [27]. For certain CVD conditions, particularly hypertension, the associations between wartime stressors and late-life cardiovascular comorbidities diverge across gender by women experiencing higher penalties for their exposure to war-related stressors than their male counterparts [28].

The epidemic of obesity in the general population has increased over the past decade, affecting 37.7% of adults [9]. In the literature, over 44% of veterans were obese (BMI > 30 kg/m^2^), which followed our results [29]. Metabolic syndrome affects about 20–30% of the general population. Its incidence increases with age in a sex-specific manner. Below the fifties, it is slightly higher in men, and it reverses after the fifties [30,31,32]. In a Croatian study, 25% of war veterans with PTSD had metabolic syndrome, and its prevalence was three times higher in patients with severe depression [33]. Worldwide, 9.3% of the population has diabetes, and in Croatia, it accounts for 7.44% [34]. The prevalence of comorbid diabetes and PTSD was 8–10% [23,24]. Some authors suggest that clinically meaningful reductions in PTSD symptoms were associated with a lower risk of T2D [35]. Increasing age was synonymous with a higher prevalence of T2DM [24]. In our cohort, after analyzing only HbA1c levels, there were 20 newly diagnosed diabetics and 34 of them with impaired glucose tolerance. In the study by Trief et al., in the male veteran’s sample, the average HbA1c was 8% [36]. We found that an average HbA1c among veterans with PTSD and DMT2 was 8%.

Although CVD can be preventable, they cause 31% of all deaths worldwide. Because of that, an urgent need for routine screening for different cardiovascular risk factors has been raised [9]. However, despite mounting evidence, PTSD has not yet been acknowledged as a risk factor by cardiovascular or endocrinological societies [37]. Pleiotropy in genetic vulnerability and pathophysiological mechanisms, such as those leading to the increased central and peripheral activation of immune metabolic or endocrine systems, plays a role in metabolic syndrome and posttraumatic stress disorder development [38].

Furthermore, knowledge about diabetes mellitus, its complications, chronic therapy, and treatment of hypoglycemia was insufficient. Reeducation should be performed regularly with disease control and drug adherence reevaluation. All these bring us to the conclusion that posttraumatic stress disorder is not the only psychiatric diagnosis. Easily performable and early targeted national screening programs and preventive check-ups directed to cardiovascular diseases prevention in individuals with PTSD are mandatory. We propose the incorporation of OSA screening as well as taking into consideration metabolic disturbances in the holistic approach to the patient with PTSD. Further studies on a larger cohort of participants analyzing all cardiometabolic risk factors are needed.

## 5. Limitations

This study was subject to several limitations. First, there was potential selection bias in veterans who chose to seek group treatment of PTSD. That is particularly relevant when considering the patients’ cooperation as well as acceptance of the disease. Both of these leads to better treatment. Second, PTSD symptoms might be over-reported by veterans for secondary gain. Third, and in line with the previous point, we did not use criterion standard diagnostic instruments to determine the presence of OSA; however, we used self-report measures. Finally, we have found that PTSD in veterans is associated with a high prevalence of OSA. While insomnia is a core feature of PTSD, OSA is another cause of poor sleep quality and is rarely considered in PTSD treatment. Results strongly support further research into early detection of OSA risk associated with PTSD. Screening for DM as well as pre-diabetes may result in better DM treatment and prevention of DM complications. On the other hand, early diagnosis and treatment of OSA should lead to better sleeping and control of PTSD. Comprehensive cardiometabolic screening should be part of standard care of war veterans with PTSD.

## 6. Conclusions

War veterans with posttraumatic stress disorder increased both OSA and DM risk. Even 95% of them had positive STOP-Bang results, and more than a half had a high risk for obstructive sleep apnea. Furthermore, 54% of our participants were unaware of having glucose abnormality and predominately those with higher risk for OSA. Forty-six and forty-eight of them were overweight and obese, respectively. Diabetes mellitus, depression, and arterial hypertension were independent predictive risk factors for positive STOP-Bang questionnaire results. In addition, their knowledge about comorbidities was insufficient. To the best of our knowledge, this was the first study to evaluate comprehensively several cardiometabolic risk factors and analyze knowledge and attitudes about the disease in the population of European veterans with PTSD. Further studies on larger cohorts are mandatory.

## Figures and Tables

**Table 1:** Medians (min-max) of anthropometric measurements for all participants divided into three group according to estimated risk of OSA through STOP-Bang score.

Group Parameter	Low-Risk of OSA N = 5	Intermediate-Risk of OSA N = 42	High-Risk of OSA N = 53	*p* *
Age, years	63.5 (50–67)	54 (43–65)	53 (40–67)	0.462
BMI, kg/m^2^	29.10 (22.40–31.60)	29.45 (20.80–54.01)	29.70 (22.20–42.80)	0.550
Neck circumference, cm	39.80 (37.00–42.50)	41.45 (37.50–49.05)	41.80 (35.70–49.20)	0.673
Waist circumference, cm	96.90 (91.20–102.90)	100.05 (44.50–136.90)	101.90 (86.10–137.80)	0.672
Waist-hip ratio	0.91 (0.89–0.94)	0.90 (0.39–1.03)	0.93 (0.80–1.12)	0.972

* Kruskal-Wallis test.

**Table 2:** Presentation of participants’ attitudes about material status, working status, and quality of sleep according to estimated OSA risk.

Group Parameter		Low-Risk of OSA N = 5	Intermediate-Risk of OSA N = 42	High-Risk of OSA N = 53	*p* *
Working status, N (%)	Retired (N = 47)	2 (40)	17 (40.48)	28 (52.83)	0.746
	Unemployed (N = 34)	1 (20)	14 (33.33)	19 (35.85)	
	Employed (N = 19)	1 (20)	7 (16.67)	11 (20.75)	
Incomes, N (%)	Much worse than average (N = 43)	2 (40)	16 (38.09)	25 (47.17)	0.832
	Worse than average (N = 20)	1 (20)	9 (21.43)	10 (18.87)	
	Average (N = 34)	2 (40)	18 (42.86)	14 (26.42)	
	Better than average (N = 3)	0 (0)	1 (2.38)	2 (3.77)	
	Much better than average (N = 0)	0 (0)	0 (0)	0 (0)	
Disrupted sleep, N (%)	N = 53	2 (40)	21 (50)	30 (55.55)	0.121
Higher daily stress, N (%)	N = 54	2 (40)	22 (52.38)	30 (55.55)	0.497
Nighmares, N (%)	N = 99	5 (100)	41 (97.62)	53 (100)	0.996
Posibility of relaxing, N (%)	Yes	2 (40)	39 (92.86)	4 (7.55)	<0.001
	No	3 (60)	3 (7.14)	49 (92.45)	
Hospitalizations during the war due to head trauma, N (%)		0 (0)	32 (76.19)	14 (26.41)	<0.001

* Chi-square test.

**Table 3:** Distribution of participants according to OSA risk stratification and diagnosis of (pre)diabetes.

Group Parameter	Low-Risk of OSA N = 5	Intermediate-Risk of OSA N = 42	High-Risk of OSA N = 53	*p*
Length of PTSD diagnosis, years	10 (1–23)	12.5 (1–27)	10 (0.17–25)	0.251 *
Participants with previously diagnosed DM, N (%)	0 (0)	0 (0)	13 (24.53)	0.011 **
Participants with newly diagnosed prediabetes, N (%)	0 (0)	8 (19.05)	26 (49.06)	<0.001 **
Participants with diagnosed DM after checkup, N (%)	0 (0)	0 (0)	20 (37.74)	<0.001 **
Measured HbA1c, %	5.5 (5.2–7.8)	5.7 (4.6–10.0)	5.6 (5.0–8.3)	<0.001 *

* Kruskal-Wallis test. ** Chi-square test.

**Table 4:** Prevalence of other cardiovascular risk factors according to OSA risk, number of participants (%).

Group Parameter	Low-Risk of OSA N = 5	Intermediate-Risk of OSA N = 42	High-Risk of OSA N = 53	*p* *
Smoking, N (%)	1 (20)	18 (42.86)	35 (66.04)	0.023
Arterial hypertension, N (%)	0 (0)	5 (11.90)	30 (56.60)	<0.001
Hyperlipidemia, N (%)	3 (60)	9 (21.43)	13 (24.53)	0.169
Hyperuricemia, N (%)	0 (0)	1 (2.38)	1 (1.89)	0.832
Chronic kidney disease, N (%)	0 (0)	0 (0)	1 (1.89)	0.741

* Chi-square test.

## Data Availability

All data are available with the corresponding author. You can contact her on e-mail: mmizdrak@mefst.hr.

## Appendix A

STOP-Bang questionnaire: 1. Do you snore loudly (loud enough to be heard through closed doors, or your bed partner elbows you for snoring at night)? 2. Do you often feel tired, fatigued, or sleepy during the daytime (such as falling asleep during driving)? 3. Has anyone observed you stop breathing or choking/gasping during your sleep? 4. Do you have or are being treated for high blood pressure? Body mass index more than 35 kg/m^2^? 5. Age older than 50 years old? 6. Is neck size large (measured around Adam’s apple)? 7. Is your shirt collar 16 inches or larger? 8. Gender (biological sex) = Male? [12,13].
